# Safety and Immunogenicity of the Candidate Tuberculosis Vaccine MVA85A in West Africa

**DOI:** 10.1371/journal.pone.0002921

**Published:** 2008-08-13

**Authors:** Roger H. Brookes, Philip C. Hill, Patrick K. Owiafe, Hannah B. Ibanga, David J. Jeffries, Simon A. Donkor, Helen A. Fletcher, Abdulrahman S. Hammond, Christian Lienhardt, Richard A. Adegbola, Helen McShane, Adrian V. S. Hill

**Affiliations:** 1 Bacterial Diseases Programme, Tuberculosis Division, Medical Research Council Laboratories, Fajara, Banjul, The Gambia; 2 Centre for Clinical Vaccinology and Tropical Medicine, University of Oxford, Churchill Hospital, Oxford, United Kingdom; 3 Institut de Recherche pour le Développement, Paris, France; 4 Wellcome Trust Centre for Human Genetics, University of Oxford, Oxford, United Kingdom; University College London, United Kingdom

## Abstract

**Background:**

Vaccination with a recombinant modified vaccinia Ankara expressing antigen 85A from *Mycobacterium tuberculosis*, MVA85A, induces high levels of cellular immune responses in UK volunteers. We assessed the safety and immunogenicity of this new vaccine in West African volunteers.

**Methods and Findings:**

We vaccinated 21 healthy adult male subjects (11 BCG scar negative and 10 BCG scar positive) with MVA85A after screening for evidence of prior exposure to mycobacteria. We monitored them over six months, observing for clinical, haematological and biochemical adverse events, together with assessment of the vaccine induced cellular immune response using ELISPOT and flow cytometry. MVA85A was well tolerated with no significant adverse events. Mild local and systemic adverse events were consistent with previous UK trials. Marked immunogenicity was found whether individuals had a previous BCG scar or not. There was not enhanced immunogenicity in those with a BCG scar, and induced T cell responses were better maintained in apparently BCG-naïve Gambians than previously studied BCG-naïve UK vaccinees. Although responses were predominantly attributable to CD4+ T cells, we also identified antigen specific CD8+ T cell responses, in subjects who were HLA B-35 and in whom enough blood was available for more detailed immunological analysis.

**Conclusions:**

These data on the safety and immunogenicity of MVA85A in West Africa support its accelerated development as a promising booster vaccine for tuberculosis.

**Trial Registration:**

ClinicalTrials.gov NCT00423839

## Introduction

Tuberculosis (TB) causes approximately 2 million deaths annually; most of these occur in developing countries[1]. The only available vaccine, BCG, is widely used in developing countries. It reduces the incidence of tuberculous meningitis and disseminated TB in childhood, but has variable efficacy against pulmonary TB[2], [3]. A major hypothesis regarding the poor efficacy of BCG vaccination in developing countries is that intense exposure to environmental mycobacteria reduces the immunogenicity and efficacy of BCG[4], [5].

We have developed a prime-boost strategy that seeks to overcome this limitation[6]. It is based on the striking effect of non-replicating poxviruses on the amplification of pre-existing T cell responses[7]–[9]. Expressing a major secreted antigen of *M. tuberculosis* (antigen 85A) in the non-replicating modified vaccinia viral vector (MVA) strongly boosts BCG primed T cell responses in several species, including humans. Importantly, BCG-MVA85A prime-boost regimens have greater protective efficacy than BCG alone in several animal models of TB, including non-human primates.[10], [11](Verreck et al, manuscript submitted)

We have reported the immunogenicity of MVA85A in UK volunteers[12], [13]. The T cell responses observed are the highest reported for any subunit vaccine against any disease. Responses were highest in those primed with BCG, but even those subjects not primed had stronger T cell responses than seen with other vaccine candidates. This is thought to relate to prior exposure to environmental mycobacteria and induction of central memory T cell responses, which are then boosted with MVA85A [12]. In view of the attenuated immunogenicity of BCG in Africa and reports that many other novel vaccines had reduced immunogenicity in developing countries compared to the country-of-origin,[14]–[16] we assessed MVA85A in The Gambia at an early stage of its clinical development. Here we report the safety and immunogenicity results from this trial.

## Methods

### Study setting and recruitment

The protocols for this trial and the supporting CONSORT checklist are available as supporting information; see Checklist S1 and Protocols S1 and S2. The study was conducted in Banjul, between 2003 and 2005, in an area housing approximately 600,000 people. The study setting and the inclusion and exclusion criteria have been described previously[17]. The study protocol (GM 920) was approved by the Gambia government/MRC (SCC 920) and Oxfordshire Tropical Research Ethics committees (OxTREC 006-03). After written informed consent, interview, and clinical examination (males aged 18 to 45 years), blood samples were collected for ELISPOT, haematology and biochemistry, and HIV and HBV antibody tests. HIV positive subjects were referred to the MRC HIV clinic, where free anti-retroviral treatment is available. Each subject had a chest X-ray read by two specialist physicians, and a PPD skin test (2TU PPD RT23, SSI, Copenhagen, Denmark) read by a trained field worker at 48–72 hours. We aimed to deliver MVA85A to volunteers with incrementally increasing evidence of prior mycobacterial exposure. The first arm of the trial enrolled BCG scar negative, and the second arm BCG scar positive, individuals. The severity of local and systemic adverse events was classified using standard criteria, as previously used in the UK studies with this vaccine.

### Vaccination and follow-up

**Figure 1:**
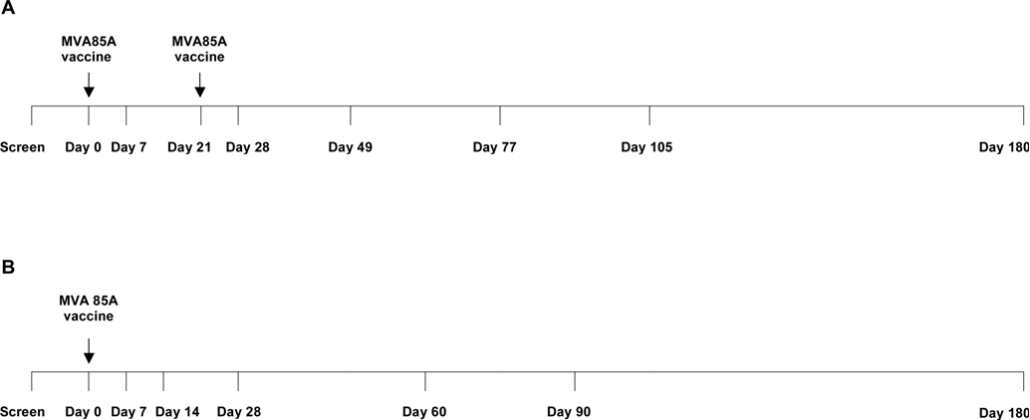
Timeline for vaccination and blood sampling schedules. A. BCG scar negative subjects. B. BCG scar positive subjects.

Those eligible were vaccinated within 8 weeks of screening. Five were vaccinated in the BCG negative arm before enrolment into the BCG positive arm began. On the vaccination day they had a clinical evaluation and provided a blood sample. The vaccine was administered intra-dermally over the insertion of the left deltoid muscle, at a dose of 5×10^7^ plaque forming units (pfu) of MVA85A (135 µl). A second vaccination was given 3 weeks later to the BCG scar negative vaccinees, in the right deltoid muscle. Subjects were observed for one hour following immunisation and vital signs were recorded. They were then seen on day one and two. Follow-up visits were made (Figure 1) to enquire about possible adverse events and any medications taken. All signs and symptoms were recorded. On each of the vaccination and follow-up days, blood was obtained for immunological assays; haematological and biochemical analyses were repeated on days 7 and 84.

### ELISPOT screen and Immunogenicity

We used an IFN-γ ELISPOT assay to screen volunteers at recruitment and monitor the immunogenicity of MVA85A, as previously described[18]. Briefly, PBMCs were plated at 3×10^5^ cells/well. Sequential peptides (15mers overlapping by 10) spanning the length of ESAT-6 and CFP-10, were used in pools at 2.5 µg/ml (ABC, Imperial College, London, UK). *M. tuberculosis* purified protein derivative (RT49, SSI, Copenhagen, Denmark; PPD-T) was used at 5 µg/ml. ELISPOT plates were incubated overnight at 37°C. For the BCG scar negative group, positive wells were pre-defined to contain at least 30 Spot Forming Units/million cells (SPM) more than, and at least twice as many as, negative control wells for these antigens. Following the UK trials, a much stronger response to vaccination was expected in the BCG primed group. Therefore, positive wells were pre-defined to contain at least 10 SPM more than, and at least twice as many as, negative control wells. After acceptable safety data were obtained in BCG scar negative group, the PPD ELISPOT screening criterion was relaxed to at least 100 SPM.

To monitor Ag85A immunogenicity we used the ELISPOT response to a sum of 66 pooled peptides (7 pools of 6–10 peptides). This method will count twice a T cell that responds to any of the 10-mer overlap regions that occur in two pools with adjacent peptides. To monitor individual peptide responses we used Ag85A peptide pools in different combinations so that an individual peptide response would be clearly identifiable when two pools with the same peptide responded within a matrix. We used recombinant Ag85A protein (Leiden University), and PPD-T. ELISPOT plates were counted using an automated ELISPOT reader (AID-GmbH, Germany) with correction for artefacts. The positive control was Phytohaemaglutinin (PHA; Sigma-Aldrich, UK). All antigens were tested in duplicate wells.

### CD4+ and CD8+ T cell activation as measured by CD69 expression

An aliquot of whole blood (200 µl) was harvested after 20 h of culture at 37°C alone or stimulated with 5.0 µg/ml Ag85A peptide pool, 2.0 µg/ml PPD-T and 10 µg/ml PHA. After incubation, cells were stained for 30 min at room temperature with a monoclonal antibody cocktail mix containing anti-CD4 FITC, anti-CD69 PE, anti-CD8 PerCP and anti-CD3 APC (BD Biosciences, UK). Red blood cells were lysed and lymphocytes washed, fixed in 2% p-formaldehyde and acquired with a Cell Quest software by live gating on CD3+ T cells (50000 events) on a four-colour flow cytometer (BD FASCalibur, BD Biosciences, UK). Analyses were carried out using FlowJo software (Treestar Inc., San Carlos, CA).

### CD8+ T cell lines

Cryopreserved PBMC were thawed, washed and re-suspended at 2×10^5^/well in a 96 U well microtitre plate with p23 peptide (10 µg/ml) in RPMI-1640 supplemented with 5% AB serum, L-glutamine 2mM and antibiotics. On the 3^rd^ and 7^th^ days IL-2 (20 IU/ml) was added. The expanded lymphocytes were harvested, washed, re-stimulated with p23 (10 µg/ml), PHA (10 µg/ml) or medium alone in separate tubes in the presence of Brefeldin A (10 µg/ml) for six hours. Cells were harvested and washed with FACs buffer (PBS, 2mM EDTA and 1%BSA) lysed with BD FACS™ Lysing Solution. Cells were then washed and permeabilized with BD FACS Permeabilizing Solution. After an additional wash, cells were divided into two tubes. Surface and intracellular staining antibodies (anti human, CD4-PE, CD69-PerCP, CD3-APC and IFN-γ -FITC) in tube 1 and anti human (CD8-PE, CD69-PerCP, CD3-APC and IFN-g-FITC) in tube 2 were added in a single staining step. Finally, the cells were washed and fixed for flow cytometric analysis.

### Peptide stimulation

The Ag85A HLA-B35 restricted CD8+ epitope MPVGGQSSF (ABC, Imperial College, London, UK ) was used to stimulate the cryopreserved PBMC isolated on day 7 after vaccination in an *ex vivo* ELISPOT assay. Recovered cells were rested in medium containing 5% human AB serum for at least 2 hours before being washed and set up in an ELISPOT assay with the CD8+ epitope.

### Data management and statistical analysis

All data were either double entered, or electronically transferred, into an ACCESS database and manually validated. ELISPOT counts were corrected for the background in the negative control well. Those beyond the upper limit of detection of the ELISPOT reader were arbitrarily given a count of 500 spots/well. All comparisons were within subject and were performed using a Wilcoxon matched pairs test.

## Results

### Screening

We screened 240 subjects to identify 10 to 12 individuals for each arm of the study. Some subjects showed transient ESAT-6/CFP-10 ELISPOT positivity after enrolment, consistent with fluctuations in responses to these antigens seen in other Gambian studies[19]. Therefore, a re-screen ELISPOT was conducted on the day before vaccination for subjects in the BCG scar positive group.

### Safety of MVA85A

Overall, 21 volunteers were enrolled and 31 doses of MVA85A were administered. One subject in the BCG scar negative group did not receive the second vaccination because he became ESAT-6 ELISPOT positive on day 7. On subsequent assays this reverted to negative. No significant changes occurred in vital signs during the one-hour post-vaccination observation or follow-up periods. There were minimal reports of any systemic side effects; specifically no symptoms of a flu-like illness or lymphadenopathy. One subject had a mild headache and diarrhoea in the first 24 hours after vaccination, both resolved quickly. No other reports or documentation of headache, myalgia, fever, or malaise were made. Table 1 shows the local signs and symptoms that were encountered. Induration at the injection site was almost universal and the majority had at least one other symptom or sign. None reported limitation of arm movements or the need to refrain from certain activities due to pain. The skin was not warmer than the surrounding regions. One volunteer still had tenderness at the site of the first vaccination at four weeks. This was elicited by firm pressure. Discolouration and scaling, when present, disappeared by day 28 in all subjects. There were no significant laboratory abnormalities. There were no differences in either the local or systemic adverse event profiles between the BCG scar positive and BCG scar negative subjects.

**Table 1:** Clinical outcomes at the injection sites after vaccination with MVA-85A.

Adverse Event	Dose 1 (*n* = 21)	Dose 2 (*n* = 10)
**Discolouration, median (range) mm**	4 (5–14)	0 (0) a
		1(3)^b^
**Pain**	11	1^a^
		2^b^
**Induration**	20	9^a^
		7^b^
**Scaling**	13	0^a^
		7^b^

The data were recorded over seven days after each vaccination

n: number of volunteers vaccinated and followed up

a first vaccination site

b residual change at the second vaccination site

### Immunogenicity of MVA85A in BCG Scar negative vaccinees

**Figure 2:**
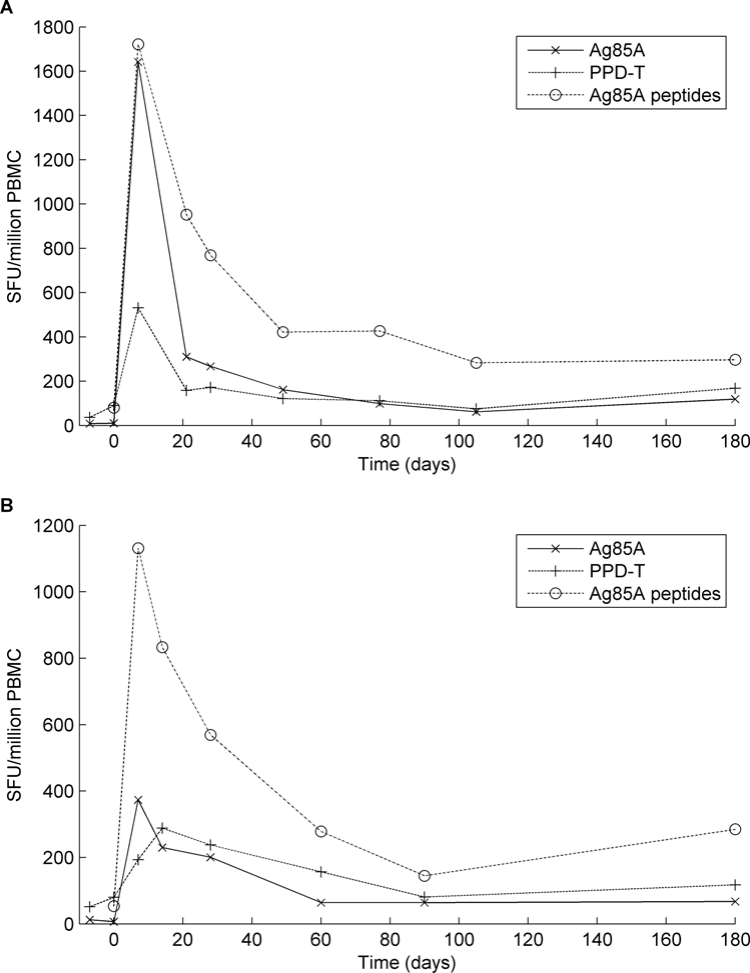
Median Ex-vivo interferon-gamma ELISPOT responses to Ag85A protein, PPD-T and Ag85A overlapping peptides after MVA85A vaccination in healthy male volunteers. A. BCG scar negative vaccines (n = 11); B. BCG scar positive vaccinees (n = 10).

11 BCG scar negative subjects were immunised with MVA85A (Figure1A). There was a high degree of variability between individuals in the vaccine induced immune responses; these data are shown as median ELISPOT frequencies in Figure 2A and the inter-quartile ranges are shown in Table 2. The ELISPOT responses one week after vaccination reached or exceeded the maximal readily quantified level for the majority (60%) of subjects. The increase in ELISPOT response at one week was highly significant for Ag85A peptides (p = 0.002), Ag85A protein (p = 0.004), and PPD-T (p<0.001).

**Table 2:** Median and inter-quartile range of ELISPOT responses for Ag85A protein, PPD-T and Ag85A overlapping peptides at each time point.

ELISPOT count (Median & inter-quartile range; SPM^a^ PBMC^b^)										
Ag85A					PPD-T			Ag85A peptides		
**BCG scar –**	***day***	***lower quartile***	***median***	***upper quartile***	***lower quartile***	***median***	***upper quartile***	***lower quartile***	***median***	***upper quartile***
	**0**	5	10	42	43	89	208	57	79	131
	**7**	296	1625	1645	236	526	1381	1001	1704	3843
	**21**	74	307	531	96	157	453	424	943	1559
	**28**	73	264	1353	77	170	472	263	761	1704
	**49**	40	160	404	76	121	162	253	418	1545
	**77**	80	97	166	78	111	166	144	422	1102
	**105**	33	61	116	49	74	121	123	281	502
	**180**	40	118	183	79	167	210	142	294	924
**BCG scar +**	**0**	3	7	26	28	79	94	26	53	89
	**7**	224	367	475	149	191	422	558	1120	2049
	**14**	99	228	647	168	286	617	515	825	1960
	**28**	43	199	348	71	235	307	92	564	1219
	**60**	35	64	264	50	155	191	228	276	661
	**90**	12	64	134	36	80	157	108	144	377
	**180**	44	67	151	88	116	183	151	282	449

a SPM, Spot Forming Units/million.

b PBMC, Peripheral Blood Mononuclear Cells

After the first week, the response contracted to a stable plateau frequency, higher than before vaccination, which was maintained until the end of the trial. The frequency of the Ag85A ELISPOT response to peptides at the end of the trial was significantly higher than the frequency at the start (p = 0.004). A consistent hierarchy of responses to antigens was found in a predictable order, from highest to lowest: summed pooled peptides, Ag85A, and PPD-T. It was not possible to accurately quantify differences at the peak of response, as many were beyond the upper limit of detection. However the original hierarchy was maintained as the response waned. A second MVA85A vaccination given 3 weeks after the first did not boost the ELISPOT response further, consistent with the UK trials [12].

### Immunogenicity in BCG Scar positive vaccinees

On the basis of the data from the BCG scar negative subjects, where there was no significant increase in the immune response to a second vaccination, it was decided that the second immunisation with MVA85A at week 3 should be omitted in the BCG scar positive group (Figure 1B). An extra visit at Day 14 was added to monitor immunogenicity. The schedules in the two groups were otherwise comparable. The profile and hierarchy of response to antigens were similar to the BCG scar negative group (data not shown): the immunogenicity of Ag85A (Figure 2B; Table 2) was similar, with most of the subjects showing a maximal response one week after vaccination (day 7). As with the BCG scar negative group, the ELISPOT frequencies were significantly higher on day 7 after vaccination than the responses at the start of the trial for Ag85A peptides (p = 0.002), Ag85A protein (p = 0.002) and PPD-T (p<0.001). Unexpectedly, three subjects achieved a maximal response at day 14, in contrast to the expected 7 day peak time point, but in the absence of a day 14 sample in the BCG scar negative group, a formal comparison was not possible. Peptide responses were broad and spanned the length of antigen 85A. Responses to peptides p9, p13, p24 and p28 were most frequently observed (data not shown), consistent with the UK trials [12].

### CD69 expression on antigen specific CD4+ and CD8+ T cells

We stimulated the whole blood of 3 volunteers with all 66 Ag85A peptides and monitored the proportion of CD4+ and CD8+ cells activated by upregulation of expression of CD69 (Table 3). A high proportion of both CD4+ and CD8+ cells were activated one week following vaccination at frequencies higher than might have been estimated from the IFN-γ ELISPOT frequencies (2,200–2,800 SPM).

**Table 3:** CD69 expression in Antigen 85A specific, CD4+ and CD8+ T cells.

	Proportion of CD69 expression		
Experiment number	Day 0	Day 7	Day 49
**CD8+ cells**			
**1**	0.00	0.22	0.56
**2**	ND	0.28	0.02
**3**	0.00	0.24	0.02
**CD4+ cells**			
**1**	0.04	0.27	1.84
**2**	ND	1.95	0.00
**3**	0.05	1.09	0.11

Frequencies of positive cells are given as a percentage of the total gated lymphocytes.

### Identification of CD8+ T cell responses

**Figure 3:**
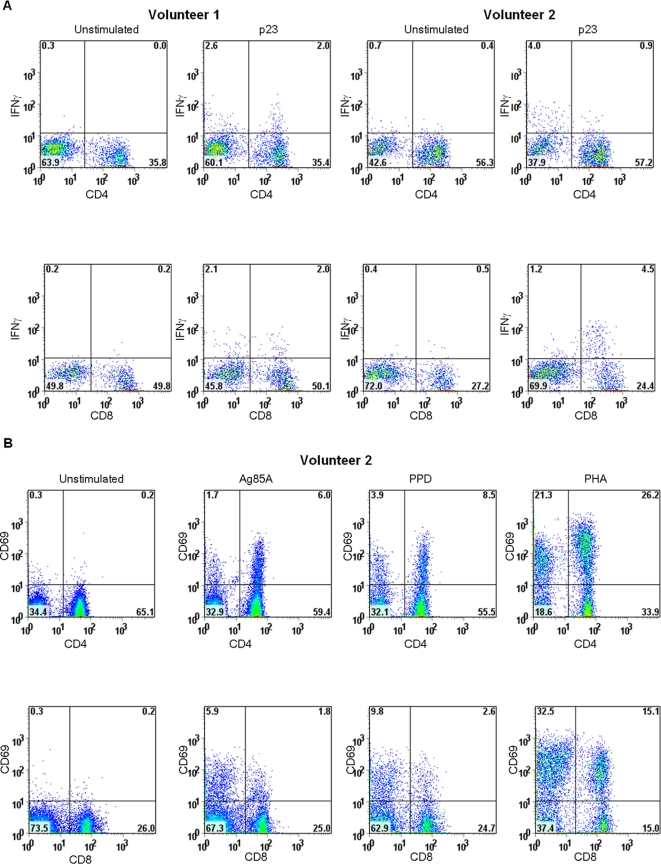
CD8+ and CD4+ T cell responses. A. Short term cell lines specific for p23 were generated using PBMC recovered 28 days after vaccination and specificity demonstrated by flow cytometry. Gating on IFN-γ detection, both CD4 and CD8 responses were observed for one subject (volunteer 1) while for the other subject (volunteer 2) an exclusive CD8 response was identified. B. Volunteer 2 also showed CD4 and CD8 activation by CD69 expression after stimulation with pooled Ag85A peptides 28 days after vaccination.

To understand responding phenotypes in more detail we looked for unambiguous peptide pool responses, consistent with a single epitope being maintained throughout the entire monitoring period. After analysing all peptide pool responses we identified a single epitope in 2 subjects, which was due in both cases to the response to a single peptide (p23). Short term cell lines to p23 were generated with PBMC recovered 28 days after vaccination, and the CD4/CD8 phenotype was analysed by IFN-γ ICS. For one subject (volunteer 1), both CD4 and CD8 p23 specific responses were identified (Figure 3A), while an exclusive CD8 response was found for the second subject (volunteer 2; Figure 3A). Volunteer 2 also showed CD8 activation by CD69 expression after stimulation with pooled Ag85A peptides 28 days after vaccination (Figure 3B).

### Confirmation of dominant HLA-B35 CD8+ T cell response

An HLA-B*3501 (abbreviated to HLA-B35) class I restricted CTL epitope within Ag85A p23 sequence had previously been reported in The Gambia[20] and HLA-B35 is also a common allele in this population[21]. We confirmed by PCR that two subjects were positive for the HLA-B35 allele. Both of these subjects responded with levels of 90 and 110 SPM, respectively, with PBMC recovered one week post vaccination, but no response prior to vaccination. The data confirm that both subjects had vaccine-induced HLA-B35 restricted CD8+ T cells specific for a previously identified Ag85A epitope.

## Discussion

We report the first trial of a new generation TB vaccine in Africa. MVA85A was administered intra-dermally to two groups of volunteers with increasing evidence of prior mycobacterial exposure. The vaccine was found to be safe in these volunteers, enabling further studies to proceed in larger numbers of individuals to fully evaluate the adverse event profile and frequency. Since safety was demonstrated in these trials in The Gambia, further trials with this vaccine in TB endemic areas in individuals more representative of the general population, including subjects latently infected with *M. tuberculosis*, have commenced. This vaccine was found to be highly immunogenic in the studies reported here, inducing exceptionally high frequencies of antigen specific CD4+ T cells as well as some antigen specific CD8+ T cells.

The peak responses to vaccination with MVA85A, in the UK and The Gambia, are the highest reported for any subunit vaccine in humans, at least using ELISPOT assays[22] [9]. The induced T cell responses are broad, target multiple epitopes and are predominantly CD4+. Although MVA85A was known to induce CD4+ T cell mediated immunity, we now show that it also induces detectable CD8+ T cell responses, in the few subjects we were able to study in detail in this trial. Since CD8+ T cells were documented by focusing on peptide responses that persist throughout the trial period, it will be important to determine whether ELISPOT responsive CD8+ T cells survive longer than their CD4+ counterparts, as has been suggested [23]. Several reports have provided evidence for some protective role of classical CD8+ T cells in TB, at least in murine models, so this is an important finding [24]–[26]. If CD8+ T cells are found to be generally more readily induced by MVA85A in Africa than the UK, we speculate that this may relate to differential priming by environmental mycobacteria in the two settings. We were only able to perform the peptide mapping in a very limited number of subjects due to restrictions in the volume of blood we were permitted to take. In addition, only two subjects were found to be HLA B35 positive, and we were therefore only able to look for responses to the B35 restricted CD8+ T cell epitope in them. Further, more detailed, work in the expanded ongoing trials is needed to confirm the presence of CD8+ T cell responses induced by MVA85A in TB endemic areas.

The lack of any boosting effect seen with the second MVA85A vaccination mirrors the findings of the UK trial.[12] It is likely that the lack of boosting at such a short interval is due to anti-vector immunity–which may be B and/or T cell mediated. Interestingly, a study in the Gambia with a recombinant MVA expressing a malaria antigen, showed that it was possible to boost responses with a second MVA to the same magnitude as the first, with an interval of one year between vaccinations.[27] This has important implications for the increasing use of this vector as a vaccine for a number of pathogens, and further work is needed to define the nature and duration of the anti-vector immunity.

Unlike the MVA85A trial in the UK,[12] there was no significant difference in the immunogenicity between BCG scar positive and BCG scar negative groups in The Gambia. It appears possible that a greater exposure of Gambians to environmental mycobacteria compensates for the lack of a BCG immunisation. Therefore, a BCG priming immunisation may be most important in young children. It should be noted that the absence of a BCG scar is not a fully reliable indicator of the lack of earlier vaccination[28], [29].

An interesting difference is observed between the plateau level of T cell response in the Gambian vaccinees and in subjects receiving the same vaccine in the UK. While the peak responses were little different, the plateau response in BCG naïve Gambians of over 200 SPM to antigen 85A peptides was higher than that observed in the UK (median 105 SPM). Whether this relates to higher pre-vaccination levels of central memory cells in Gambians, or more frequent low level boosting by recurrent exposure to low doses of environmental mycobacteria in Africa, requires further study.

Whilst we went to significant efforts to ensure that the first volunteers were as mycobacterially naïve as possible, it is likely that most if not all individuals had been exposed to mycobacteria before[30]. The discovery of a transient ESAT-6/CFP-10 ELISPOT response was unexpected but suggests there is a consistent low background of mycobacterial exposure in the community. The PPD skin test is a particularly sensitive, and surprisingly specific, indicator of recent *M. tuberculosis* infection in The Gambia [31], [32] and it is possible that some of the transient ESAT-6/CFP-10 response may be due to an uncharacterised environmental mycobacterial infection–certain environmental mycobacteria, such as *M. marinum,* express these two antigens [33], [34] and are commonly encountered in The Gambia.

In conclusion, MVA85A appears safe and highly immunogenic in Africa. The data from this study support accelerated development of this candidate towards a large scale efficacy trial in Africa and subsequent licensure. Further phase I and II trials are now in progress in a South African population and a phase II trial in Gambian infants is also underway.

## Supporting Information

Checklist S1 Consort Checklist.
(0.06 MB DOC)

Protocol S1 Trial Protocol.
(0.21 MB PDF)

Protocol S2 Trial Protocol.
(0.21 MB PDF)
