# Correction: Safety and Immunogenicity of the Candidate Tuberculosis Vaccine MVA85A in West Africa

**DOI:** 10.1371/annotation/5284a0b6-62a9-484e-80a4-a08c59b3b17c

**Published:** 2011-02-26

**Authors:** Roger H. Brookes, Philip C. Hill, Patrick K. Owiafe, Hannah B. Ibanga, David J. Jeffries, Simon A. Donkor, Helen A. Fletcher, Abdulrahman S. Hammond, Christian Lienhardt, Richard A. Adegbola, Helen McShane, Adrian V. S. Hill

The following information was missing from the Funding section: "The study was supported by funding from the NIHR Oxford Biomedical Research Centre programme."

