# Supplementary material for: Safety and Immunogenicity of the Candidate Tuberculosis Vaccine MVA85A in West Africa
Source: PLoS One. 2008 Aug 13;3(8):e2921. doi: 10.1371/journal.pone.0002921 (PMC2488375; doi:10.1371/journal.pone.0002921)
Supplement: Checklist S1 — Consort Checklist. (0.06 MB DOC) [file pone.0002921.s001.doc]

# CONSORT Statement 2001 - Checklist
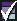


**Items to include when reporting a randomized trial**

| ***PAPER SECTION* And topic** | Item | **Descriptor** | **Reported** |
| --- | --- | --- | --- |
| TITLE & ABSTRACT | 1 | [How participants were allocated to interventions](http://www.consort-statement.org/index.aspx?o=1107) (*e.g*., "random allocation", "randomized", or "randomly assigned"). | Methods |
| *INTRODUCTION* Background | 2 | [Scientific background and explanation of rationale](http://www.consort-statement.org/index.aspx?o=1016). | Intro & Methods |
| *METHODS* Participants | 3 | [Eligibility criteria for participants](http://www.consort-statement.org/index.aspx?o=1017#3a) and the [settings and locations where the data were collected](http://www.consort-statement.org/index.aspx?o=1017#3b). | Methods |
| Interventions | 4 | [Precise details of the interventions intended for each group and how and when they were actually administered](http://www.consort-statement.org/index.aspx?o=1021). | Methods |
| Objectives | 5 | [Specific objectives and hypotheses](http://www.consort-statement.org/index.aspx?o=1022). | Intro & Methods |
| Outcomes | 6 | [Clearly defined primary and secondary outcome measures](http://www.consort-statement.org/index.aspx?o=1023#6a) and, when applicable, any [methods used to enhance the quality of measurements](http://www.consort-statement.org/index.aspx?o=1023#6b) (*e.g.*, multiple observations, training of assessors). | Methods |
| Sample size | 7 | [How sample size was determined](http://www.consort-statement.org/index.aspx?o=1024#7a) and, when applicable, [explanation of any interim analyses and stopping rules](http://www.consort-statement.org/index.aspx?o=1024#7b). | Methods |
| Randomization -- Sequence generation | 8 | [Method used to generate the random allocation sequence, including details of any restrictions](http://www.consort-statement.org/index.aspx?o=1025) (*e.g*., blocking, stratification) | NR |
| Randomization -- Allocation concealment | 9 | [Method used to implement the random allocation sequence](http://www.consort-statement.org/index.aspx?o=1026) (*e.g*., numbered containers or central telephone), clarifying whether the sequence was concealed until interventions were assigned. | NR |
| Randomization -- Implementation | 10 | [Who generated the allocation sequence, who enrolled participants, and who assigned participants to their groups](http://www.consort-statement.org/index.aspx?o=1027). | NR |
| Blinding (masking) | 11 | [Whether or not participants, those administering the interventions, and those assessing the outcomes were blinded to group assignment](http://www.consort-statement.org/index.aspx?o=1028#11a). If done, [how the success of blinding was evaluated](http://www.consort-statement.org/index.aspx?o=1028#11b). | NR |
| Statistical methods | 12 | [Statistical methods used to compare groups for primary outcome(s)](http://www.consort-statement.org/index.aspx?o=1029#12a); [Methods for additional analyses](http://www.consort-statement.org/index.aspx?o=1029#12b), such as subgroup analyses and adjusted analyses. | Methods |
| RESULTS Participant flow | 13 | [Flow of participants through each stage](http://www.consort-statement.org/index.aspx?o=1018) (a diagram is strongly recommended). Specifically, for each group report the numbers of participants randomly assigned, receiving intended treatment, completing the study protocol, and analyzed for the primary outcome. [Describe protocol deviations from study as planned, together with reasons](http://www.consort-statement.org/index.aspx?o=1086). | See separate diagram |
| Recruitment | 14 | [Dates defining the periods of recruitment and follow-up](http://www.consort-statement.org/index.aspx?o=1087). | Methods |
| Baseline data | 15 | [Baseline demographic and clinical characteristics of each group](http://www.consort-statement.org/index.aspx?o=1088). | Reference 17 |
| Numbers analyzed | 16 | [Number of participants (denominator) in each group included in each analysis and whether the analysis was by "intention-to-treat"](http://www.consort-statement.org/index.aspx?o=1089). State the results in absolute numbers when feasible (*e.g*., 10/20, not 50%). | Results |
| Outcomes and estimation | 17 | [For each primary and secondary outcome, a summary of results for each group, and the estimated effect size and its precision](http://www.consort-statement.org/index.aspx?o=1090) (*e.g.*, 95% confidence interval). | NR |
| Ancillary analyses | 18 | [Address multiplicity by reporting any other analyses performed](http://www.consort-statement.org/index.aspx?o=1091), including subgroup analyses and adjusted analyses, indicating those pre-specified and those exploratory. | NR |
| Adverse events | 19 | [All important adverse events or side effects in each intervention group](http://www.consort-statement.org/index.aspx?o=1092). | Results |
| *DISCUSSION* Interpretation | 20 | [Interpretation of the results](http://www.consort-statement.org/index.aspx?o=1019), taking into account study hypotheses, sources of potential bias or imprecision and the dangers associated with multiplicity of analyses and outcomes. | Discussion |
| Generalizability | 21 | [Generalizability (external validity) of the trial findings](http://www.consort-statement.org/index.aspx?o=1094). | Discussion |
| Overall evidence | 22 | [General interpretation of the results in the context of current evidence](http://www.consort-statement.org/index.aspx?o=1095). | Discussion |

**www.consort-statement.org**
