# Supplementary material for: Safety and Immunogenicity of the Candidate Tuberculosis Vaccine MVA85A in West Africa
Source: PLoS One. 2008 Aug 13;3(8):e2921. doi: 10.1371/journal.pone.0002921 (PMC2488375; doi:10.1371/journal.pone.0002921)
Supplement: Protocol S1 — Trial Protocol. (0.21 MB PDF) [file pone.0002921.s002.pdf]

African Tuberculosis Vaccine Trial (AFTBVAC)

A phase I study of the safety and immunogenicity of MVA85A in healthy Gambian volunteers

SCC number 920

Principal Investigators: Roger Brookes, Philip Hill

Study Protocol

Hannah Ibanga

February, 2003

Team Roster

Principal Investigators

Dr Roger Brookes
MRC, The Gambia
Ext: 359
Email: rbrookes@mrc.gm

Dr Philip Hill
MRC, The Gambia
Ext 402
Email: phill@mrc.gm

Co-Investigators

Dr Hannah Ibanga
Dr Annette Fox
Dr Samuel McConkey
Mr Patrick Owiafe
Dr Dolly Jackson-Sillah
Dr Katherine Fielding
Dr David Jefferies
Dr Tumani Corrah
Dr Kebba Manneh
Prof Adrian Hill
Dr Sarah Gilbert
Dr Helen McShane
Dr Helen Fletcher

International EU Grant collaborators
Dr Christian Lienhardt
Prof Souleymane
Andreas Neubert
Dr Kris Huygen

Field Researchers
Aziz Bangura

PARTICIPATING SITE

MRC Labs, Fajara, The Gambia

Protocol Statistician

Dr Katherine Fielding
LSTHM
Katherine.Fielding@lshtm.ac.uk

Data Manager and database designer

Simon Donkor
Ext: 360
Email: Sdonkor@mrc.gm

Dr David Jeffries
Ext:
Email: Djeffries@mrc.gm

Data entry: Yandeh Jobe, Roger Badgie

Table of Contents

Study Management **4**

Schema **5**

1.0 INTRODUCTION 6

2.0 STUDY OBJECTIVES 8

3.0 SELECTION AND ENROLLMENT OF SUBJECTS 8

4.0 CLINICAL AND LABORATORY EVALUATIONS 11

5.0 DATA COLLECTION AND MONITORING AND ADVERSE EXPERIENCE REPORTING 10

6.0 STATISTICAL CONSIDERATIONS 12

7.0 HUMAN SUBJECTS 12

8.0 PLANS FOR DISTRIBUTION OF RESEARCH FINDINGS TO STUDY COMMUNITY 13

9.0 BIOHAZARD CONTAINMENT 13

APPENDICES 14

Study Management

All questions concerning this protocol should be sent via e-mail to hibanga@mrc.gm.

For Questions About Inclusion/Exclusion Criteria, The Schedule Of Events, Case Report Forms, Entering Waivers For Randomization/Registration Exemptions, Transfers, And Delinquencies, And Other Data Management Issues, the Project Data Manager will respond:

Send an e-mail message to phill@mrc or rbrookes@mrc.gm
Include the MRC number and household number
Give a detailed description of the question

For Adverse Events Questions:

Send an e-mail message to hibanga@mrc.gm or
Call MRC ext 359

Schema

TITLE: A phase I study of the safety and immunogenicity of MVA85A in healthy Gambian volunteers

LOCATED: MRC Labs, Fajara, The Gambia

ESTIMATED COMPLETION DATE: June 30, 2004

DESIGN: A dose-escalating open label non-randomised Phase I trial of a new TB subunit vaccine.

SAMPLE SIZE: 24 subjects

POPULATION: Subjects will be adults 18 to 45 years of age.

OBJECTIVE: To assess the safety and immunogenicity of a recombinant MVA vaccine expressing the tuberculosis antigen 85A, MVA85A, in healthy Gambian volunteers.

1.0 INTRODUCTION

1.1 Background

The huge scale of global TB mortality, the increasing prevalence of HIV infection and the spread of multi-drug resistant TB, along with the variable efficacy of BCG against pulmonary TB, has led to renewed efforts to develop effective TB vaccines. Two types of approach are being developed, the use of genetically modified mycobacteria and subunit vaccines. The former approach may be some way from clinical testing and there are major regulatory hurdles to the deployment of such vaccines, so much TB vaccine research and development now focuses on new subunit vaccines. These will have to work by stimulating the cellular arm of the immune system. The field of induction of T cell mediated protection by subunit vaccines is a new one, but important for the development of new vaccines for malaria, HIV and cancers as well as TB. CD4 T cells that activate macrophages are of central importance in TB immunity as illustrated by the increased susceptibility of HIV positive individuals to TB. Evidence from gene knockout mice, adoptive transfer experiments in mice and human correlative analyses also support a protective role for classical CD8 T cells in both mice and humans. Thus new TB subunit vaccines should aim to induce both CD4 and CD8 T cell responses. Current subunit vaccines divide into those that efficiently boost pre-existing T cell responses, such as MVA, fowlpox and adenovirus vectors and those that do not, such as DNA, lipopeptides and particles. Recombinant proteins that are non-particulate generally induce no, or very weak, CD8 T cell responses whatever adjuvant is used, because they fail to access intracellular class I processing pathways. In the developing world a large proportion of healthy individuals have T cell responses to mycobacteria because they are latently infected with Mtb or other mycobacteria, and Mtb infected individuals are at increased risk of clinical tuberculosis compared to non-infected. These individuals require a post-exposure vaccine that will enhance their immune responses and prevent disease. For these there is a good case for using two boosting vectors, such as FP9 and MVA, rather than just one (the MVA in DNA-MVA) in heterologous prime-boost immunisation strategies. Differences between the immunogenicity and protective efficacy of BCG vaccine in Europe and Africa make a strong case for early assessment of new subunit vaccines in Africa. Immunogenicity studies have recently demonstrated that most immunised Malawians had pre-existing anti-PPD T cell responses that were not enhanced further by BCG vaccination, in marked contrast to the UK vaccinees in which BCG vaccine was highly immunogenic. Prior exposure to environmental mycobacteria in Africa probably pre-sensitises most individuals to mycobacterial antigens and BCG vaccine then fails to generate or boost Mtb-specific responses, probably because its replication is impaired. BCG is also ineffective as a therapeutic vaccine in animals. In contrast to BCG, the MVA and FP9 viral vectors should enhance anti-mycobacterial responses in Africans because of their excellent boosting abilities and their capacity to strongly boost immune responses post-BCG immunisation, thereby providing the basis for a post-exposure TB vaccine. But clearly this key question needs to be assessed directly. The importance of early African studies of prime-boost vaccine regimes has been demonstrated in malaria vaccine trials. Higher immunogenicity of DNA-MVA malaria vaccines was observed in Africans compared to using the same vaccines in Europeans. Notably these Africans were previously malaria-exposed, providing evidence that MVA can boost pre-existing T cells responses (both CD4 and CD8) in Africans.

Secreted antigens from *M. tuberculosis* are released from actively metabolising bacteria, and are important targets in protective immunity. Antigen 85A is a major secreted antigen from *M. tuberculosis* which forms part of the antigen 85 complex (A, B and C). This complex constitutes a major portion of the secreted proteins of both *M.tb* and BCG. It is involved in fibronectin binding within the cell wall and has mycolyltransferase activity. Dr Huygen and colleagues have cloned and characterised this antigen and demonstrate that it is a protective antigen in small animal TB challenge models, particularly when used as a

DNA vaccine. MVA85A induces both a CD4+ and a CD8+ epitope when used to immunise mice. When mice are primed with BCG and then given MVA85A as a boost, the levels of CD4+ and CD8+ T cells induced are higher than with either BCG or MVA85A alone. The Oxford co-investigators have extended this work to studies in guinea pigs with prime-boost immunisation regimes in which Ag85A was protective, to macaques where both MVA85A and FP9-85A were strongly immunogenic. In Oxford phase I clinical studies with the same viral vector are currently being carried out. These latter clinical trials in Europe seek to evaluate initially and sequentially the safety and the immunogenicity of BCG, MVA85A and FP9-85a used individually and in heterologous prime-boost regimes. Initially, non-mycobacterially infected individuals (skin-test and ELISPOT negative) are being studied, which is to be followed by studies of healthy infected contacts.

1.2 Rationale

An effective TB vaccine for use in the developing world must be of low-cost and have high stability. The recombinant MVA vaccine can be manufactured economically to high titre in a GMP manufacturing facility. Costs of manufacturing poxviruses are substantially lower that DNA vaccines on a dose per dose basis and these viruses may be freeze dried for transport without a cold chain.

The parental vaccinia virus was once used as a smallpox vaccine (MVA) was previously deployed world-wide to successfully eliminate smallpox and was not associated with serious adverse effects. MVA appears to be an exceptionally safe viral vector and was used recently without inducing significant adverse events even in severely immunocompromised macaques. Over 400 people, including over 250 Gambians adults and children, have now been immunised with various recombinant MVA investigational vaccines including constructs expressing malaria, HIV, hepatitis B and melanoma antigens without significant adverse events.

1.3 Study Design

The study is a non-randomized clinical trial. Twelve volunteers will be recruited, who will be healthy individuals with no evidence of mycobacterium infection. They would be given $5x10^7$ pfu of the MVA85A vaccine intradermally twice at three weekly intervals. The subjects will be required to stay in the unit for an hour after each vaccination. Local and systemic adverse events would be recorded. Blood samples will be taken at the screening visit, both days of immunisation, 1 week after each vaccination, then at 4, 8, 12 and 24 weeks after the second vaccination. If the vaccine were shown to be safe, a higher dose ($1x10^8$pfu) of the vaccine would be administered to a second group of twelve healthy volunteers who have no evidence of mycobacterium infection. At the 6 month follow-up visit, subjects will be immunized with BCG and blood taken and followed up at 1,4 and 12 weeks.

2.0 STUDY OBJECTIVES

1. To assess the safety and immunogenicity of a recombinant MVA vaccine expressing the tuberculosis antigen 85A, MVA85A, in healthy Gambian volunteers.

3.0 SELECTION AND ENROLLMENT OF SUBJECTS

3.1 Inclusion Criteria

- Healthy adult male aged 18-45 years (no history of BCG vaccination).

- Normal medical history and physical examination. Minor physical ailments e.g. Fungal skin infections, will not be sufficient to define a physical examination as abnormal.
- Normal urine dipstick, blood count, liver enzymes, and creatinine.
- Frequency less than 10 SFC per/well/2x10^5 PBMC as determined by ELISPOT with ESAT6/CFP-10 antigens and PPD.
- Mantoux negative (0.0 mm induration).
- Normal Chest X-ray.
- Willing to donate blood samples as required by the protocol
- Negative HIV antibody test or no serological evidence of HCV or HBV infection.

3.2 Exclusion criteria

- Definite or probable exposure to BCG vaccination at any point.
- Clinically significant history of skin disorder (eczema, psoriasis, etc.), allergy, immunodeficiency, cardiovascular disease, respiratory disease, endocrine disorder, liver disease, renal disease, gastrointestinal disease or neurological illness.
- Any clinical evidence of immunosuppression such as oral candida, stomatitis, aphthous or septic ulceration, septic skin lesions or any clinical or laboratory evidence of infection or immunocompromise.
- History of splenectomy
- Haematocrit of less than 30%
- Serum creatinine concentration >130mmol
- Serum ALT concentration >80IU/L
- Blood transfusion within one month of the beginning of the study
- History of vaccination with any previous experimental poxvirus vaccine
- Administration of any other vaccine or immunoglobulin within two weeks before or two weeks after vaccination.
- Positive HIV antibody test, evidence of HBV or HCV disease (Hepatitis B vaccination is not an exclusion criterion).
- Current participation in another clinical trial, or within 12 weeks of this study
- Any other finding which in the opinion of the investigators would increase the risk of an adverse outcome from participation in the trial.
- Likelihood of travel away from the study area for the duration of the study
- Untreated malaria infection

3.3 Enrollment Procedures

Subjects will be recruited from at the healthy relatives of patients at the MRC Gate or Outpatient Clinic, MRC staff and their relatives, external controls from the current TBCC study, healthy blood donors at the Royal Victoria Hospital and youth groups of mosques and churches, football clubs. The purpose of the study would be described to the prospective volunteers and what it involves for participants. At the at the MRC the nature of the trial will be fully explained including the methods of vaccination, observation details, spectrum of likely side effects, follow-up details and extent of blood sampling. Volunteers must understand that these vaccines have not yet been shown to prevent tuberculosis and this will be stressed during the recruitment stage.

3.4 Substudies

Substudies will take place; these will be all covered in the consent forms.

3.5 Co-enrollment Guidelines:

As is MRC policy, study subjects are encouraged not to be involved in other studies. Certainly they should not be subjects in a vaccine trial or any drug trial.

4.0 CLINICAL AND LABORATORY EVALUATIONS

4.1 Pre-entry/Entry Evaluations

4.11 Screening

Details of the study will be carefully discussed with the subjects, who will be asked to read and sign (or thumb-print) an informed consent approved by the combined MRC/Gambian government ethics committee prior to any study-related evaluations being performed. The subjects that agree to enroll and have signed consent documentation will be assessed at the AFTBVAC clinic at MRC as described below. At enrollment the consent forms are to be completed.

4.12 Entry

Subjects will be recruited at the MRC AFTBVAC clinic. Subjects will be screened in the eight weeks prior to entering the study. The screen will consist of checking subject eligibility and a full physical examination. The following will be carried out: height, weight, vital signs, haematology, serum chemistry, Mantoux test, ELISPOT, Chest X-ray, anti-vaccinia antibodies, anti-HBV antibodies, anti-HCV antibodies, anti-HIV antibodies, urinalysis.

4.2 Evaluations During study

4.21 Scheduled follow-up

First Vaccination: Day 0: On each vaccination day subjects will visit the clinical site. The following assessments will be performed pre-dosing: vital signs (20 minutes pre-dose). Provided all is satisfactory, subjects will receive the first vaccination. Subjects will have a dressing applied over the injection site, which will remain for at least one hour after the vaccination. They will remain at the clinical area for one hour following vaccination and will then be allowed to return home.

Day 1 and 2: On the first and second day after vaccination subjects will be visited at home by a field worker or they will return to the clinical area. The injection site will be examined and the subjects will be questioned for AEs.

Day 7: Subjects will be visited at home by a field worker or they will return to the clinical area. Vital sign assessments will be performed. Blood samples will be taken for haematology, serum chemistry and immunological assays. The injection site will be examined and the subjects will be questioned for AEs.

Second Vaccination: Day 21: Twenty one days after vaccination subjects will return to the clinical area. Vital sign assessments will be performed. Blood samples will be taken prior to dosing for haematology,

serum chemistry and immunological assays. The injection site will be examined and the subjects will be questioned for AEs. Subjects will receive the next vaccination. Subjects will have a dressing applied over the injection site and left for at least one hour after the vaccination. They will remain at the clinical site for one hour following vaccination and will then be allowed to return home.

Day 22 and 23: On the first and second day after vaccination subjects will be visited at home by a field worker or they will return to the clinical area. The injection site will be examined and the subjects will be questioned for AEs.

Day 28,49, 77, 105 and 180: Subjects will return to the clinical site for clinical assessment and blood samples will be collected for haematology, serum chemistry and immunological assays. The injection site will be examined and the subjects will be questioned for AEs.

4.3 Subjects who prematurely discontinue the study

Subjects who leave the study at any time, may do so without repurcussions. They may still access medical care at the MRC, and will not be turned away.

4.4 Post study Evaluations:

Subjects will be given BCG at the end of the study and seen on week 1,4 and12 when blood samples will be taken for heamatology, serum chemistry and immunological assays. The injection site will be examined and the subjects asked about AEs. It is possible that they will be asked in the future to provide samples for genetic studies.

5.0 DATA COLLECTION AND MONITORING AND ADVERSE EXPERIENCE REPORTING

5.1 Records to Be Kept

A regulatory folder will contain:

- SCC document
- Project Management plan
- Protocol with appendices
- Letter of ethical approval
- Information sheets and blank forms
- Signed consent forms for each subject

A protocol deviations folder will contain documentation of all pre-planned deviations from the protocol and their justification.

A protocol violations folder will contain documentation of unplanned protocol violations.

All filled in paper forms will be filed. Individual medical records will be filed together.

All data on the Case Report Forms (CRF) must be legibly recorded in blue or black ink or typed. A correction should be made by striking through the incorrect entry with a single line and entering the correct information adjacent to it. The correction must be initialed and dated by the investigator or a designated, qualified individual.

Any requested information that is not obtained as specified in the protocol should have an explanation noted on the CRF as to why the required information was not obtained.

5.2 Role of Data Management

The Data manager will be responsible for receiving, entering, cleaning, querying, analysing, and storing the data which accrues from the study. He will be responsible for linking the epidemiological and clinical data from the field and the clinic with the laboratory data from the immunology, microbiology, haematology and genetics laboratories.

5.4 Serious Adverse Experience (SAE) Reporting

The MRC health and safety manager, the chairman of the SCC, and the chairman of the ethics committee, will be informed of any adverse event of any kind.
Adverse events, however minor, will be recorded as observed by the Investigators or as volunteered by the subject. Full details will be documented in the CRF whether or not the Investigator or his deputies consider the event to be related to the trial substance.
Serious adverse events (SAEs) that occur during the study or within six months of the final vaccination will be notified immediately by telephone to the Ethics Committees that have approved the project.
Serious adverse event are defined as an event that:

- results in death;
- is life-threatening (*i.e.,* the subject was at risk of death at the time of the event);
- requires or prolongs in-patient hospitalization;
- results in persistent or significant disability/incapacity;
- is a congenital anomaly/birth defect;
- is a cancer.

Minimum details to be given in a telephone report are:

- Name of reporting doctor and contact telephone number.
- Study number.
- Nature of adverse event.
- Subject details (number, initials, sex, date of birth, weight and age).
- Date and time of event.
- Date and time of MVA85A administration and dose.
- Other drug history.
- Other relevant history.
- Outcome.
- Causality.

The event will be documented on the SAE page of the CRF and reported to the Regulatory Authorities as appropriate. Other adverse events will be graded according to Appendix 4. If any SAE occurs then that volunteer will not be administered further vaccinations. After the ethics committee's response to the SAE is received, the Principal Investigator, Clinical Monitor and available co-investigators will meet to determine the future plan for the study, which could involve amending the protocol, discontinuing the vaccinations, or continuing unchanged for the other volunteers.

6.0 STATISTICAL CONSIDERATIONS

6.1 General Design Issues

A total of 24 subjects who will be divided into 2 groups of 12 will be sufficient to provide descriptive data. As some of the subjects may drop out of the study, 12 subjects i.e. 12/ group will be recruited. However, only 10 individuals per group will be given the vaccine.

6.2 Outcomes of interest

6.21 Safety of the Vaccine

This will be determined by the degree and number of adverse events reported.

6.22 Immunogenicity of this vaccine

It is expected that the vaccine will stimulate T cell responses, which will be measured by interferon –gamma Elispot assays.

6.3 Sample Size and Accrual

Formal sample sizing calculations have not been performed but it is believed that the sample size of 10 subjects per group will be sufficient for this purpose.
No attempt will be made in the study to conceal the allocation group of the subjects either from the subjects themselves, the investigators or laboratory personnel.

6.4 Monitoring and Analysis

The data manager will be responsible for monitoring the trial. This will include confirming the existence of the appropriate documents in the regulatory folder and of source documents for all entered data. He would also assess the consistency of data, missing data, and abnormal data and generate queries to be addressed by the investigators. The data manager will be responsible for data entry, data cleaning and for initial analysis of the results.

The main analyses will be descriptive and comparative.

7.0 HUMAN SUBJECTS

7.1 Institutional Review Board (IRB) Review and Informed Consent

The study has been approved by the joint MRC/ Gambia government ethical committee. Written informed consent will be obtained from the subject (or parent, legal guardian, or person with power of attorney for subjects who cannot consent for themselves, such as, those below the legal age). The subject's assent must also be obtained if he or she is able to understand the nature, significance and risks associated with the study. The informed consent will describe the purpose of the study, the procedures to be followed, and the risks and benefits of participation. A copy of the consent form will be given to the subject (or parent or legal guardian).

7.2 Subject Confidentiality

All records will be kept in a secure place. All data on computer files will have restricted access. Clinical information will not be released without written permission of the subject, except as necessary for monitoring.

7.3 Study Discontinuation

The study may be discontinued by the MRC SCC or the combined MRC/Gambian government ethics committee.

8.0 PLANS FOR DISTRIBUTION OF RESEARCH FINDINGS TO STUDY COMMUNITY

At the end of the study, a research report of the methods, detailed results, and brief conclusions will be prepared for distribution to the collaborators. A simplified lay document will be made available to study subjects.

9.0 BIOHAZARD CONTAINMENT

As the transmission of HIV and other blood-borne pathogens can occur through contact with contaminated needles, blood, and blood products, appropriate blood and secretion precautions will be employed by all personnel in the drawing of blood and shipping and handling of all specimens for this study, according to the MRC safety manual.

APPENDICES

APPENDIX 1-EQUIPMENT FOR FIELDWORK

A. Recruitment team Staff

- Field worker supervisor for pre-recruitment visit
- Recruitment team leader
- Field researchers x 2
- Genetics field researcher

B. Transport

- Landrover x 1 with driver
- Motorbikes- field worker supervisor, genetics fieldworker, Mantoux reading

C. Equipment

- Bags to carry equipment
- Two cold boxes
- Ice packs
- Sharp container
- Alcohol swabs, cotton swabs, tissue papers
- Scales for weighing
- MVA85A and PPD vials
- Note books and all forms
- Syringes and needles
- Mobile phone
- Drugs
- Tubes for blood samples
- Address of subjects
- Height measuring equipment
- Resuscitation box containing Ambu bag and face masks, endotracheal tubes, oropharyngeal airways, laryngoscopes and blades, batteries
